# Into the third decade: eleventh ISABS Conference on Forensic and Anthropological Genetics and Mayo Clinic Lectures in Individualized Medicine

**DOI:** 10.3325/cmj.2019.60.189

**Published:** 2019-06

**Authors:** Stanimir Vuk-Pavlović, Moses Schanfield, Dragan Primorac

**Affiliations:** 1 Mayo Clinic College of Medicine and Science, Rochester, MN, USA; 2 George Washington University at MVC, Washington, DC, USA; 3 St. Catherine Specialty Hospital, Zagreb/Zabok, Croatia; 4 Eberly College of Science, Pennsylvania State University, State College, Pennsylvania, PA, USA; 5 School of Medicine, University of Split, Split, Croatia; 6 School of Dental Medicine and Health, Josip Juraj Strossmayer University of Osijek, Osijek, Croatia; 7 School of Medicine, Josip Juraj Strossmayer University of Osijek, Osijek, Croatia; 8 Faculty of Medicine, University of Rijeka, Rijeka, Croatia; 9 Henry C. Lee College of Criminal Justice and Forensic Sciences, University of New Haven, West Haven, CT, USA; 10 Children’s Hospital Srebrnjak, Zagreb, Croatia *draganprimorac2@gmail.com*

This year our conference is twenty-two years old – its parents proudly boast! Looking back to the first conference, also in Split, one cannot but be grateful that the initial idea took hold and gave the conference its current specific flavor (1-4). Somewhat bold and experimental at the time, the idea was to present the cutting-edge forensic, anthropological, and clinical genome-based disciplines, not on the basis of the then disparate applications, but on the commonly used technology (5,6).

This year’s conference and this issue of the *Croatian Medical Journal* offer a particularly rich program. The 11th conference will feature some seventy lecturers from institutions that include Mayo Clinic, Harvard Medical School, Columbia University, Memorial Sloan Kettering Cancer Center, University of Pennsylvania, Massachusetts Institute of Technology, Princeton University, Duke University, Max-Planck-Institute, Case Western Reserve University, Pennsylvania State University, The Wistar Institute, George Washington University, Erasmus University Medical Center Rotterdam, Seoul National University College of Medicine, University of New Haven, Nanchang University, and others. More then five hundred participants from more than forty countries will discuss cutting-edge topics in personalized medicine, forensic genetics, and molecular anthropology.

It is not out of character for the conference, always with a strong graduate education intent, to open with preconference events. These are, concurrently, the 7th Croatian Congress of Human Genetics (organized by the Croatian Society of Human Genetics, International Society of Applied Biological Sciences [ISABS], and the Croatian Society for Precision [ie, personalized] Medicine) and the Mayo Clinic Short Course on Epigenomics. The conference closes with the short course entitled “Investigation and Forensic Analysis of Fire and Explosion” given by the forensic science legend Henry Lee. The educational impact is strengthened by the participation of Nobel Prize laureates Ada Yonath, Avram Hershko, Robert Huber, and Paul Modrich; they share not just their seminal scientific contributions, but also personal experience in science and life.

The five-day conference continues the tradition of working in the plenum. Avoiding parallel sessions is in keeping with the idea that the three areas of focus merge. The merger has become evident not only through the employment of analogous or identical genetic and (epi)genomic techniques, but also by sessions and lectures that reach across these disciplines. For the full program, the reader is referred to the ISABS Conferences webpage (*www.isabs.net*). This year, the American Academy of Forensic Sciences and ISABS jointly organize an interdisciplinary session underlining the importance of proper interpretation of scientific data in court. In addition, the future of personalized medicine is the topic both of the ISABS/Regiomed Kliniken/University of Split joint session and of the Philips Personalized Medicine Session, while St. Catherine Hospital and OneOme, the entity co-founded by Mayo Clinic, will demonstrate the importance of actionable pharmacogenetic testing to increase access to pharmacogenetic testing in Europe.

This issue of the *Croatian Medical Journal* continues the history of special issues started with the first conference. So far, these issues have published some 120 original scientific articles cited almost 2500 times, on average 20 citations per article, a considerable achievement. Along with the established practice of presenting some conference articles, this issue includes population studies (genetic polymorphism in Chinese Mongolians; population study of thrombophilic markers in Bosnia and Herzegovina; two studies of sex determination by acetabular and sternum osteometry in the Croatian population), studies in regenerative medicine (*ex vivo* gene transfer to enhance bone healing; use of lipoaspirate in the treatment of knee osteoarthritis), a genetic analysis of *CFTR* gene demonstrating the presence of del. F508 mutation and a rare combining deletion and insertion mutation p. Tyr109Glyfs in a patient with cystic fibrosis, and a case report that highlights the potential of pharmacogenomics in therapeutic decision-making while using tyrosine kinase activity inhibitors in patients with chronic myeloid leukemia.

We are truly indebted to program directors: Manfred Kayser (Erasmus University Medical Center, Rotterdam) and Tamás Ördög (Mayo Clinic, Rochester, MN) for providing the cutting-edge and fresh program this year again. Collaboration with Mayo Clinic for more than sixteen years, with the American Academy of Forensic Sciences, and others has never been stronger, leading to the ever increasing depth and breadth of the program.

Without the support and commitment of sponsors ISABS 2019 would not be possible. Particularly we thank the Croatian Tourist Board; Croatian Ministries of Tourism, Health, Science and Education as well as that of Economy, Entrepreneurship and Crafts of the Republic of Croatia; Croatian Chamber of Economy; City of Split; County of Split and Dalmatia; Municipality of Podstrana; City of Solin; Privredna banka (Bank of Commerce) Zagreb; Belupo Pharmaceuticals; Podravka Foods; Arona Hospital; Auto Benussi; OneOme LLC; Hrvatska pošta (Croatian Postal Service); Ballograf AB; Građa d.d., and Bauerfeind d.o.o; and Philips as the strategic partner of ISABS. Traditionally, the conference is held under the auspices of the Croatian Academy of Arts and Sciences and in partnership with the Croatian Medical Chamber.
